# Association of Dietary Acid Load With Metabolic Syndrome and Its Components in Iranian Adults: A Cross-Sectional Study

**DOI:** 10.7759/cureus.50593

**Published:** 2023-12-15

**Authors:** Seyede Hamide Rajaie, Reza Homayounfar, Sayyed Saeid Khayyatzadeh, Shiva Faghih, Yaser Mansoori, Mohammad Mehdi Naghizadeh, Mojtaba Farjam, Hassan Mozaffari-Khosravi

**Affiliations:** 1 Department of Nutrition, School of Public Health, Shahid Sadoughi University of Medical Sciences, Yazd, IRN; 2 Nutrition and Food Security Research Center, Shahid Sadoughi University of Medical Sciences, Yazd, IRN; 3 National Nutrition and Food Technology Research Institute, Faculty of Nutrition and Food Technology, Shahid Beheshti University of Medical Sciences, Tehran, IRN; 4 Noncommunicable Diseases Research Center, Fasa University of Medical Sciences, Fasa, IRN; 5 Department of Community Nutrition, School of Nutrition and Food Sciences, Shiraz University of Medical Sciences, Shiraz, IRN

**Keywords:** triglycerides, hdl, metabolic syndrome, neap, pral, dietary acid load

## Abstract

Introduction

Metabolic syndrome (MetS) remains one of the leading health challenges worldwide. A combination of genetic and environmental factors has been implicated in the etiology of MetS. Diet is a changeable environmental risk factor, and dietary modifications could significantly reduce the incidence and mortality of numerous diseases, including MetS. Certain dietary factors may contribute to MetS by affecting the acid-base balance within the body. This study examined the association of dietary acid load (DAL) with MetS and its components in Iranian adults.

Materials and methods

This cross-sectional study was conducted in 2022 on 6356 Iranian adults aged 35-70 years. Potential renal acid load (PRAL) and net endogenous acid production (NEAP) as two indicators of DAL were calculated based on nutrient intake data from validated food frequency questionnaires. MetS and its components were defined according to the Adult Treatment Panel III criteria. Logistic regression analysis was used to explore the associations between DAL and MetS and its components. Age, energy intake, physical activity, education, marital status, home ownership, socioeconomic status, history of obesity-related disease, and calcium supplements were included in model I. Further adjustment in model II was made for body mass index.

Results

Higher NEAP scores were associated with increased odds of low high-density lipoprotein cholesterol (HDL-C) in the crude model (OR: 1.26, 95% CI: 1.01-2.56, p trend = 0.06) in women, which was confirmed in the adjusted models.

In model I, women in the last quintile of NEAP had 54% greater odds of having hypertriglyceridemia compared to the first quintile (OR: 1.54, 95% CI: 1.007-2.36, p trend = 0.02). This association was still significant and even stronger after further adjustment for BMI (OR: 1.55, 95% CI: 1.01-2.40, p trend = 0.01). In addition, in model I, men in the fourth quintile of NEAP had 5.68-fold greater odds of hyperglycemia compared to the first quintile (OR: 5.68, 95% CI: 1.18-27.25, p trend = 0.11). Similar results were found in the fully adjusted model (OR: 5.89, 95% CI: 1.19-28.99, p trend = 0.54).

Conclusion

There was no significant association between DAL and MetS. DAL was positively associated with the odds of low HDL-C and hypertriglyceridemia in women. Moreover, moderate DAL (NEAP) was associated with an increased odds of hyperglycemia in men.

## Introduction

Metabolic syndrome (MetS) is a collection of multiple metabolic abnormalities, such as hypertension, hyperglycemia, abdominal obesity, and atherogenic dyslipidemia. It remains one of the leading health challenges worldwide, affecting 25% of all adults according to the International Diabetes Federation definition. The syndrome contributes to a 1.5-fold increase in mortality risk and imposes an enormous burden on healthcare systems [1].

A combination of genetic and environmental factors has been implicated in the etiology of MetS. Diet is a changeable environmental risk factor, and dietary modifications could significantly reduce the incidence and mortality of numerous diseases, including MetS [2]. Increasing evidence shows that mild chronic metabolic acidosis may cause high cortisol production and therefore may be related to the development of MetS [3]. Certain dietary factors have been shown to affect the body’s acid-base balance [4]. Although compensatory physiological responses in the body maintain acid-base balance closely, adherence to diets rich in acid-forming factors (such as animal products, rice, and cheese) can decrease blood pH toward the lower limit of the normal physiological range [5]. Purportedly alkaline dietary factors (such as fruits and vegetables) or homeostatic mechanisms do not compensate for this low limit of the acid-base balance. In this situation, mild chronic metabolic acidosis can result [6].

Although mild chronic metabolic acidosis might be related to the development of metabolic abnormalities, numerous acid-forming foods (such as fish, nuts, eggs, and whole grains) are rich sources of beneficial compounds, including protein, unsaturated fatty acids (omega-3, eicosapentaenoic acid, docosahexaenoic acid, and monounsaturated fatty acid), vitamins (vitamins E, B6, and D, folic acid, riboflavin, and niacin), minerals (iron, zinc, calcium, magnesium, potassium, and copper), and bioactive compounds [7-9]. In addition, whole grains provide mechanical advantages (primarily for the gastrointestinal tract) arising from the fiber content [8]. Moreover, some dietary factors (such as butter and sugar) with negative effects on metabolic health have an approximately neutral effect on acid-base balance [10].

Given the inconsistent results of previous studies on the association between dietary acid load (DAL) and MetS [11-16] and the different assumptions on the direction of this association, the present study aimed to examine the association between DAL and MetS and its components in a large sample of Iranian adults.

## Materials and methods

Study population

Baseline data from the Prospective Epidemiological Research Studies in IrAN (PERSIAN) cohort study branch in Fasa city were used for this cross-sectional study. As part of the PERSIAN multicenter cohort study, the Fasa cohort study follows 10,138 adults aged 35-70 years old living in the Sheshdeh area of Fasa (Sheshdeh town and 24 villages surrounding it) in Fars province, Iran. The detailed protocol for the Fasa PERSIAN cohort study is available elsewhere [17].

Potential participants were selected through multistage cluster random sampling and recruited after they had provided written informed consent. Blood samples and information on general characteristics, demographic status, anthropometric indices, dietary intakes, and lifestyle-related factors were collected from eligible participants. All data were collected during a face-to-face interview with a pretested questionnaire [17].

Data for 10,138 participants were included in the present study. Participants with missing data for dietary intake (n = 20) or outcomes of interest (n = 15), as well as those who under- or over-reported calorie intake (<800 kcal/d or >4200 kcal/d; n = 1278), were excluded. In addition, pregnant and lactating women, and participants with a history of diseases such as kidney failure, diabetes, or hypertension were excluded from the study because of possible dietary modifications (n = 2429). Moreover, participants with a body mass index (BMI) of 40 or higher were excluded because they might under-report their dietary intakes (n = 40). Thus, 6356 individuals were included in the final analysis.

Dietary assessment

In face-to-face interviews with trained dietitians, the usual food intakes during the previous year were obtained using a validated block-format 125-item semi-quantitative food frequency questionnaire (FFQ) [18]. As a part of the interview, participants were asked to report their average food intake frequency per day, week, or month based on household measurements. Then portion sizes of food items were converted to grams. The energy and nutrient contents of foods were derived from the US Department of Agriculture nutrient database modified for Iranian foods [19].

Net endogenous acid production (NEAP) and potential renal acid load (PRAL) calculation

Two scores are used to estimate DAL: NEAP and PRAL. The NEAP score was developed by Frassetto et al. [4] and is based on total dietary protein intake and potassium intake. Low NEAP scores mean that the diet has lower acid-forming potential, while high scores indicate higher acid-forming potential. The PRAL score is another validated tool introduced by Remer and Manz [20]. Estimates are derived based on average intestinal absorption rates of ingested total protein, as well as potassium, calcium, phosphorus, and magnesium. Diets with negative or positive PRAL scores tend to have base-forming and acid-forming properties, respectively [4,20]. The following formulas were used to calculate these scores:

NEAP (mEq/d) = 54.5 × protein g/d/potassium (mEq/d) − 10.2.

PRAL (mEq/d) = (0.49 × protein (g/d)) + (0.037 × phosphorus (mg/d)) − (0.021 × potassium (mg/d)) − (0.026 × magnesium (mg/d)) − (0.013 × calcium (mg/d)).

Anthropometric and blood pressure measurements

Weight was measured to the nearest 0.1 kg with a digital scale, and height was measured to the nearest 0.5 cm with a measuring tape, while participants wore light indoor clothing. BMI was calculated as body weight (kg) divided by height squared (m2). Waist circumference (WC) was measured after participants removed their clothing and exhaled normally, with no pressure applied to the body, at a distance halfway between the iliac crest and the lowest rib [21]. To measure blood pressure with a mercury sphygmomanometer, participants were asked to rest for 10 minutes. Two measurements were taken from the right arm at 15-minute intervals, and the average of both measures was recorded.

Biochemical assays

Blood samples were collected after participants refrained from eating for 12-14 hours, and were stored at −70°C. Triglycerides (TG), fasting blood sugar (FBS), and high-density lipoprotein cholesterol (HDL-C) were measured with an autoanalyzer system (Selectra E, Vitalab, Holliston, the Netherlands) and Pars Azmoon kits (Tehran, Iran).

Socioeconomic status (SES)

The wealth score index (WSI) represents SES, estimated by multiple correspondence analysis of the following variables: access to a freezer, washing machine, dishwasher, computer, internet, motorcycle, car (no access, access to a car costing <11,000 US dollars, or access to a car costing >11,000 US dollars), vacuum cleaner, and color television (no color television or regular color television vs. plasma color television). Other variables included in the WSI are possessing a mobile phone, a personal computer, or a laptop, and having traveled abroad (never, pilgrimage only, both pilgrimage and non-pilgrimage trips) [17].

Assessment of other variables

A pretested questionnaire was used in face-to-face interviews to collect information on age (continuous), sex (male/female), education (graduated from university vs. no university education), marital status (married vs. single or divorced), home ownership (owner vs. non-owner), active smoking (currently smoking at least one cigarette a day), previous diagnoses of fatty liver disease (yes/no), depression (yes/no) or thyroid disorder (yes/no), and use of calcium supplements (with or without vitamin D) (yes/no). The participants were questioned about their regular physical activity during the previous year, and their physical activity level was expressed as metabolic equivalent hours per week (METs h/w) [22].

Definition of MetS

MetS was defined according to Adult Treatment Panel III criteria. The syndrome is considered to exist when at least three of the following components are present: (1) abdominal obesity (WC > 102 cm for men and >88 cm for women); (2) high serum TG levels (>150 mg/dL); (3) low serum HDL-C levels (<50 mg/dL for women and <40 mg/dL for men); (4) elevated blood pressure (systolic blood pressure (SBP) ≥ 130 mm Hg and/or diastolic blood pressure (DBP) ≥ 85 mm Hg); and (5) abnormal glucose homeostasis (FBS ≥ 110 mg/dL) [23].

Statistical analysis

To assess differences in variables across quintiles of NEAP and PRAL scores, one-way ANOVA (quantitative variables) and chi-squared tests (qualitative variables) were used [24]. Dietary intakes across quintiles of NEAP and PRAL scores were compared with analysis of covariance (ANCOVA) to adjust for energy intake (kcal/d) and age.

Binary logistic regression with adjusted models was used to calculate the odds ratios (OR) and 95% confidence intervals (CI) for MetS and its components across quintiles of NEAP and PRAL scores in men and women. The first quintile group was used as the reference for ORs and their 95% CI estimates [25]. Model I was adjusted for the effects of age, energy intake (kcal/d), physical activity (continuous), education, marital status, home ownership, SES, history of obesity-related disease (fatty liver disease, depression, and thyroid disease), and use of calcium supplements (with or without vitamin D). Further adjustment for BMI was used in model II. All statistical analyses were done with SPSS version 21 (IBM Corp., Armonk, NY). P-values were considered significant at <0.05.

## Results

After eligibility was verified according to the inclusion criteria, data for 6356 participants (mean age: 46.58 ± 8.82 years, mean BMI: 25.02 ± 4.60 kg/m2) were included in the present analysis. MetS was prevalent in 777 (12.2%) participants.

The general characteristics of men and women across quintiles of NEAP and PRAL scores are shown in Table 1. Men in the highest PRAL quintile were more likely to have thyroid disease (p = 0.02) compared to men in the lowest quintile. In women, those in the highest PRAL quintile, compared to those in the lowest quintile, were more likely to be homeowners (p = 0.04) and less likely to have fatty liver disease (p = 0.004) and to have central obesity (0.03). In addition, women in the lowest NEAP quintile were more likely to have higher SES (p = 0.01) and fatty liver disease (p = 0.001) and to have central obesity (p = 0.01) than those in the highest quintile.

**Table 1:** Characteristics of the participants across quintiles of PRAL and NEAP Note: Data are presented as mean ± standard error or absolute number (percentage). Abbreviations: PRAL, potential renal acid load; NEAP, net endogenous acid production; METs h/w: metabolic equivalent hours per week. ^a^ Obtained from one-way ANOVA or chi-squared tests, as appropriate. ^b^ General obesity was defined according to cutoff values established by the WHO (obesity: BMI ≥ 30 kg/m2). ^c^ Central obesity was defined according to the Adult Treatment Panel III (ATP III) criteria (waist circumference >102 cm for men and >88 cm for women). ^d^ Socioeconomic status is presented as the wealth score index (WSI). ^e^ Self-reported.

	Quintiles of PRAL					p-value^a^	Quintiles of NEAP					p-value^a^
Variables	1	2	3	4	5		1	2	3	4	5	
Men												
Range		-23.81, -12.95	-12.95, -5.15	-5.13, 4.19	>4.19		<29.77	29.77, 37.26	37.30, 44.32	44.33, 53.52	>53.52	
n	637	638	638	638	638		569	643	644	658	675	
Age (years)	47.95 ± 0.36	47.31 ± 0.35	47.00 ± 0.35	47.16 ± 0.35	46.80 ± 0.35	0.19	47.72 ± 0.39	47.53 ± 0.35	47 ± 0.35	46.86 ± 0.35	47.18 ± 0.34	0.41
General obesity (%)^b^	51 (8)	57 (8.9)	46 (7.2)	50 (7.8)	37 (5.8)	0.29	41 (7.2)	64 (10)	46 (7.1)	46 (7)	44 (6.5)	0.14
Central obesity (%)^c^	63 (9.9)	56 (8.8)	58 (9.1)	57 (8.9)	55 (8.6)	0.94	49 (8.6)	63 (9.8)	58 (9)	59 (9)	60 (8.9)	0.96
Marital status (married) (%)	621 (97.5)	622 (97.5)	617 (96.7)	621 (97.3)	610 (95.6)	0.23	554 (97.4)	626 (97.4)	621 (96.4)	639 (97.1)	651 (96.4)	0.75
Smoking status (active smoker) (%)	342 (53.7)	349 (54.7)	362 (56.7)	357 (56)	351 (55)	0.84	305 (53.6)	362 (56.3)	359 (55.7)	377 (57.3)	358 (53)	0.49
Education (academic degree) (%)	21 (3.3)	27 (4.2)	24 (3.8)	25 (3.9)	30 (4.7)	0.76	23 (4)	29 (4.5)	26 (4)	21 (3.2)	28 (4.1)	0.80
Socioeconomic status^d^	0.76 ± 0.09	0.49 ± 0.09	0.50 ± 0.09	0.56 ± 0.09	0.48 ± 0.09	0.22	0.76 ± 0.10	0.58 ± 0.09	0.61 ± 0.09	0.40 ± 0.08	0.48 ± 0.09	0.10
Physical activity (METs h/w)	46.37 ± 0.56	45.83 ± 0.58	46.37 ± 0.57	44.79 ± 0.55	45.70 ± 0.57	0.27	46.09 ± 0.59	46.31 ± 0.58	45.85 ± 0.59	45.85 ± 0.56	45.01 ± 0.53	0.54
Homeownership (owner) (%)	575 (90.3)	586 (91.8)	587 (92)	583 (91.4)	585 (91.7)	0.81	517 (90.9)	585 (91)	590 (91.6)	609 (92.6)	615 (91.1)	0.81
Calcium supplementation (with or without vitamin D) (yes) (%)	54 (18.4)	54 (19.6)	64 (21)	43 (14.8)	38 (14.1)	0.12	49 (18.1)	62 (21.1)	53 (17.3)	48 (16.9)	41 (14.7)	0.37
Fatty liver disease (yes) (%)^e^	17 (2.7)	24 (3.8)	16 (2.5)	18 (2.8)	9 (1.4)	0.13	13 (2.3)	26 (4)	15 (2.3)	19 (2.9)	11 (1.6)	0.08
Depression (yes) (%)^e^	20 (3.1)	22 (3.4)	17 (2.7)	16 (2.5)	16 (2.5)	0.80	20 (3.5)	21 (3.3)	17 (2.6)	15 (2.3)	18 (2.7)	0.68
Thyroid disease (yes) (%)^e^	13 (2)	22 (3.4)	11 (1.7)	6 (0.9)	19 (3)	0.02	14 (2.5)	18 (2.8)	14 (2.2)	8 (1.2)	17 (2.5)	0.34
Women												
Range		-25.76, -14.92	-14.89, -6.63	-6.53, 1.90	>1.90		<29.77	29.80, 37.26	37.31, 44.32	44.33, 53.52	>53.54	
n	633	634	633	634	633		702	628	628	613	596	
Age (years)	45.74 ± 8.28	45.87 ± 8.64	46.26 ± 8.63	45.66 ± 8.50	46.01 ± 8.74	0.74	46.16 ± 0.32	45.59 ± 0.34	45.72 ± 0.32	46.25 ± 0.35	45.79 ± 0.35	0.58
General obesity (%)^b^	136 (21.5)	144 (22.7)	140 (22.1)	136 (21.5)	119 (18.8)	0.49	156 (22.2)	138 (22)	144 (22.9)	126 (20.9)	111 (18.6)	0.37
Central obesity (%)^c^	451 (71.2)	460 (72.6)	439 (69.4)	412 (56)	449 (70.9)	0.03	508 (72.4)	447 (71.2)	444 (70.7)	394 (64.3)	418 (70.1)	0.01
Marital status (married) (%)	525 (82.9)	536 (84.5)	514 (81.2)	528 (83.3)	522 (52.5)	0.61	584 (83.2)	523 (83.3)	521 (83)	498 (81.2)	499 (83.7)	0.81
Smoking status (active smoker) (%)	18 (2.8)	22 (3.5)	24 (3.8)	22 (3.5)	20 (3.2)	0.90	24 (3.4)	14 (2.2)	23 (3.7)	25 (4.1)	20 (3.4)	0.45
Education (academic degree) (%)	13 (2.1)	11 (1.7)	8 (1.3)	11 (1.7)	9 (1.4)	0.83	15 (2.1)	11 (1.8)	10 (1.6)	10 (1.6)	6 (1)	0.62
Socioeconomic status^d^	-0.36 ± 0.06	-0.45 ± 0.06	-0.40 ± 0.06	-0.50 ± 0.06	-0.44 ± 0.06	0.69	-0.40 ± 0.06	-0.27 ± 0.06	-0.42 ± 0.06	-0.61 ± 0.06	-0.45 ± 0.07	0.012
Physical activity (METs h/w)	38.31 ± 0.27	38.93 ± 0.28	38.46 ± 0.26	38.76 ± 0.26	38.93 ± 0.26	0.36	38.53 ± 0.27	38.63 ± 0.26	38.58 ± 0.26	38.52 ± 0.26	39.17 ± 0.27	0.41
Homeownership (owner) (%)	563 (88.9)	563 (88.8)	576 (91.0)	590 (93.1)	579 (91.5)	0.04	625 (89)	567 (90.3)	567 (90.3)	563 (91.8)	549 (92.1)	0.29
Calcium supplementation (with or without vitamin D) (yes) (%)	136 (39.7)	136 (42.4)	134 (41.9)	121 (37.8)	124 (39.2)	0.75	150 (38.1)	132 (41.4)	134 (41.7)	112 (39.4)	123 (40.7)	0.85
Fatty liver disease (yes) (%)^e^	80 (12.6)	74 (11.7)	84 (13.3)	48 (7.6)	58 (9.2)	0.004	90 (12.8)	74 (11.8)	83 (13.2)	44 (7.2)	53 (8.9)	0.001
Depression (yes) (%)^e^	57 (9)	60 (9.5)	51 (8.1)	57 (9)	46 (7.3)	0.63	67 (9.5)	59 (9.4)	49 (7.8)	53 (8.6)	43 (7.2)	0.51
Thyroid disease (yes) (%)^e^	97 (15.3)	74 (11.7)	83 (13.1)	81 (12.8)	64 (10.1)	0.07	100 (14.2)	84 (13.4)	75 (11.9)	76 (12.4)	64 (10.7)	0.37

The age- and energy-adjusted intakes of selected foods and nutrients across NEAP and PRAL quintiles in men and women are shown in Tables 2, 3, respectively. Men and women in the highest NEAP quintile had higher intakes of grains, meats, carbohydrates, total energy, protein, fat, cholesterol, folate, phosphorus, calcium, sodium, a higher sodium-to-potassium ratio, and lower intakes of dairy products, fruits, vegetables, dietary fiber, vitamin B12, potassium, and magnesium compared to those in the lowest quintile. Men and women in the highest PRAL quintile had greater intakes of grain, meat, total energy, protein, fat, cholesterol, folate, phosphorus (in men), calcium, sodium, and a higher sodium-to-potassium ratio, as well as lower intakes of vegetables, fruits, dairy products, carbohydrate, vitamin B12, phosphorus (in women), magnesium, and potassium compared to the lowest quintile (p < 0.001 for all).

**Table 2:** Dietary intakes of the participants across quintiles of NEAP scores Note: Data are presented as mean ± standard deviation. ^a^ NEAP = net endogenous acid production. ^b^ Calculated with multivariate analysis of covariance (ANCOVA). All variables except energy were adjusted for both energy intake and age. Energy was adjusted for age.

	Men					p-value^b^	Women					p-value^b^
	Quintiles of NEAP^a^						Quintiles of NEAP					
Models	1	2	3	4	5		1	2	3	4	5	
Range	<29.77	29.77, 37.26	37.30, 44.32	44.33, 53.52	>53.52		<29.77	29.80, 37.26	37.31, 44.32	44.33, 53.52	>53.54	
n	569	643	644	658	675		702	628	628	613	596	
Food groups (g/d)												
Grains	435.99 ± 162.33	547.24 ± 180.07	608.88 ± 187.28	687.46 ± 200.85	788.91 ± 224.05	<0.001	419.51 ± 166.56	521.08 ± 170.05	576.24 ± 176.67	665.13 ± 203.38	776.17 ± 239.49	<0.001
Meats	69.49 ± 41.39	81.03 ± 44.14	94.36 ± 52.92	94.37 ± 57.58	98.95 ± 61.53	<0.001	58.44 ± 34.47	74.66 ± 43.47	77.62 ± 46.00	86.00 ± 52.73	84.86 ± 62.47	<0.001
Dairy products	207.33 ± 161.08	216.28 ± 155.65	221.46 ± 166.19	187.44 ± 142.49	154.19 ± 120.99	<0.001	214.62 ± 152.66	229.03 ± 177.26	205.42 ± 165.69	199.97 ± 155.68	149.59 ± 119.54	<0.001
Fruit	526.54 ± 359.24	440.15 ± 282.06	366.70 ± 225.16	286.78 ± 180.62	198.92 ± 127.37	<0.001	525.04 ± 357.10	414.19 ± 266.13	329.20 ± 205.73	279.20 ± 178.23	189.04 ± 131.07	<0.001
Vegetable	685.63 ± 337.12	555.35 ± 256.05	495.63 ± 209.66	426.75 ± 191.66	304.66 ± 145.34	<0.001	701.27 ± 377.40	572.21 ± 250.79	474.89 ± 203.73	426.55 ± 195.61	295.96 ± 145.16	<0.001
Nutrients												
Energy (kcal/d)	2514.33 ± 728.30	2637.30 ± 715.42	2747.08 ± 703.44	2803.64 ± 696.74	2888.41 ± 694.30	<0.001	2373.47 ± 761.37	2536.54 ± 711.88	2550.67 ± 688.43	2740.78 ± 710.77	2799.46 ± 751.98	<0.001
Carbohydrate (g/d)	437.90 ± 138.88	445.09 ± 126.21	451.56 ± 123.67	461.40 ± 122.60	472.60 ± 127.09	<0.001	410.98 ± 141.81	422.68 ± 124.84	418.92 ± 120.03	446.08 ± 125.58	461.47 ± 130.82	<0.001
Protein (g/d)	72.55 ± 23.36	82.11 ± 24.62	90.04 ± 25.10	93.89 ± 26.96	99.27 ± 25.44	<0.001	68.90 ± 23.39	79.88 ± 23.99	82.98 ± 24.85	91.23 ± 25.81	95.20 ± 27.46	<0.001
Fat (g/d)	60.68 ± 22.05	64.71 ± 22.41	69.13 ± 23.17	67.86 ± 21.23	67.85 ± 22.35	<0.001	58.47 ± 22.50	64.31 ± 24.04	64.58 ± 21.63	68.84 ± 23.28	64.68 ± 21.69	<0.001
Fiber (g/d)	31.79 ± 12.10	30.08 ± 10.46	29.19 ± 9.53	27.84 ± 8.99	25.63 ± 9.96	<0.001	31.47 ± 12.44	29.67 ± 10.38	27.31 ± 9.32	27.26 ± 8.90	25.14 ± 7.96	<0.001
Cholesterol (mg/d)	225.59 ± 121.98	254.45 ± 131.72	286.60 ± 146.76	273.82 ± 140.97	275.10 ± 149.27	<0.001	198.64 ± 106.28	237.67 ± 126.08	236.52 ± 118.42	264.21 ± 143.78	244.81 ± 151.43	<0.001
Vitamin B12 (µg/d)	7.34 ± 0.16	6.93 ± 0.15	6.82 ± 0.15	5.89 ± 0.15	4.98 ± 0.15	<0.001	7.04 ± 0.15	6.37 ± 0.16	5.76 ± 0.16	5.67 ± 0.16	4.28 ± 0.16	<0.001
Folate (µg/d)	720.22 ± 5.55	719.67 ± 5.19	734.82 ± 5.18	756.18 ± 5.13	785.04 ± 5.08	<0.001	693.19 ± 5.09	697.06 ± 5.34	710.67 ± 5.33	721.29 ± 5.42	764.04 ± 5.51	<0.001
Phosphorus (mg/d)	1188.9 ± 387.61	1267.85 ± 389.43	1337.80 ± 377.68	1335.15 ± 371.92	1333.62 ± 341.79	<0.001	1148.69 ± 385.94	1236.58 ± 376.29	1227.61 ± 376.31	1304.16 ± 371.46	1284.48 ± 366.68	<0.001
Potassium (mg/d)	4507.48 ± 1438.82	3989.28 ± 1189.10	3763.11 ± 1056.49	3398.52 ± 972.71	2871.79 ± 803.01	<0.001	4373.59 ± 1493.07	3891.64 ± 1164.39	3482.00 ± 1048.83	3313.62 ± 950.30	2753.81 ± 837.64	<0.001
Calcium (mg/d)	1042.06 ± 387.83	1176.12 ± 394.49	1285.50 ± 414.46	1364.32 ± 447.18	1488.98 ± 478.19	<0.001	1019.46 ± 395.94	1175.99 ± 384.82	1227.35 ± 425.92	1362.05 ± 460.86	1485.36 ± 513.64	<0.001
Magnesium (mg/d)	386.48 ± 117.53	377.14 ± 111.32	378.56 ± 106.68	366.10 ± 99.55	346.89 ± 87.87	<0.001	372.84 ± 128.24	365.08 ± 108.03	348.66 ± 100.75	355.06 ± 99.13	336.35 ± 93.65	<0.001
Sodium (mg/d)	3808.35 ± 1385.88	4151.80 ± 1378.33	4486.92 ± 1453.65	4562.95 ± 1341.75	4887.86 ± 1402.78	<0.001	3819.26 ± 1661.86	4222.49 ± 1449.05	4259.33 ± 1461.06	4705.45 ± 1518.34	4827.30 ± 1430.30	<0.001
Sodium/potassium	0.88 ± 0.33	1.07 ± 0.32	1.22 ± 0.36	1.38 ± 0.33	1.76 ± 0.48	<0.001	0.90 ± 0.32	1.12 ± 0.40	1.25 ± 0.34	1.45 ± 0.40	1.82 ± 0.52	<0.001

**Table 3:** Dietary intakes of the participants across quintiles of PRAL scores Note: Data are presented as mean ± standard deviation. ^a^ PRAL = potential renal acid load. ^b^ Calculated with multivariate analysis of covariance (ANCOVA). All variables except energy were adjusted for both energy intake and age. Energy was adjusted for age.

	Men					p-value^b^	Women					p-value^b^
	Quintiles of PRAL^a^						Quintiles of PRAL					
Models	1	2	3	4	5		1	2	3	4	5	
Range		-23.81, -12.95	-12.95, -5.15	-5.13, 4.19	>4.19			-25.76, -14.92	-14.89, -6.63	-6.53, 1.90	>1.90	
n	637	638	638	638	638		633	634	633	634	633	
Food groups (g/d)												
Grains	517.34 ± 186.04	557.47 ± 208.25	598.42 ± 212.29	642.04 ± 205.49	784.18 ± 222.09	<0.001	490.86 ± 184.94	516.30 ± 197.52	553.24 ± 200.53	613.02 ± 209.69	753.59 ± 241.49	<0.001
Meats	80.14 ± 45.48	80.56 ± 46.80	78.83 ± 47.87	90.40 ± 54.28	111.10 ± 63.83	<0.001	67.60 ± 40.16	69.95 ± 41.68	69.67 ± 44.23	73.90 ± 45.75	97.72 ± 64.54	<0.001
Dairy products	239.65 ± 177.29	207.53 ± 157.44	194.40 ± 149.77	173.69 ± 130.39	167.98 ± 126.68	<0.001	254.25 ± 181.41	208.82 ± 151.84	197.67 ± 159.32	174.14 ± 146.60	168.06 ± 130.56	<0.001
Fruit	606.00 ± 358.98	404.01 ± 235.75	312.47 ± 182.10	257.79 ± 166.01	210.25 ± 137.77	<0.001	611.66 ± 366.67	389.83 ± 221.11	312.64 ± 186.91	247.80 ± 163.15	205.21 ± 142.95	<0.001
Vegetable	741.32 ± 316.99	536.80 ± 229.71	457.03 ± 185.82	385.74 ± 174.26	314.21 ± 157.53	<0.001	787.66 ± 373.80	564.13 ± 215.71	453.99 ± 184.15	388.97 ± 175.37	312.01 ± 160.69	<0.001
Nutrients												
Energy (kcal/d)	2853.97 ± 684.92	2631.29 ± 737.98	2589.82 ± 722.81	2619.17 ± 706.13	2930.94 ± 670.92	<0.001	2738.77 ± 724.65	2462.08 ± 725.50	2448.14 ± 724.12	2493.68 ± 725.71	2818.73 ± 729.92	<0.001
Carbohydrate (g/d)	497.02 ± 126.26	443.03 ± 127.57	429.75 ± 126.66	427.79 ± 121.81	473.99 ± 118.79	<0.001	475.01 ± 133.05	412.54 ± 125.09	404.50 ± 124.09	406.91 ± 125.77	456.97 ± 127.84	<0.001
Protein (g/d)	85.64 ± 24.83	82.28 ± 26.35	83.29 ± 25.97	86.86 ± 26.42	102.26 ± 25.35	<0.001	81.57 ± 24.93	77.25 ± 25.34	78.25 ± 25.81	81.53 ± 25.35	97.11 ± 27.37	<0.001
Fat (g/d)	66.98 ± 22.13	64.45 ± 22.70	63.67 ± 21.92	65.09 ± 22.16	70.79 ± 22.29	<0.001	66.03 ± 22.42	61.40 ± 23.20	61.59 ± 22.72	62.80 ± 22.94	68.27 ± 22.44	<0.001
Fiber (g/d)	36.33 ± 11.10	29.40 ± 9.26	26.88 ± 8.77	25.39 ± 8.36	26.02 ± 7.84	0.000	36.65 ± 11.73	28.64 ± 8.94	26.23 ± 8.76	24.63 ± 8.33	25.27 ± 8.05	0.000
Cholesterol (mg/d)	253.09 ± 130.92	253.77 ± 139.37	244.65 ± 130.05	262.08 ± 136.34	307.21 ± 155.62	<0.001	225.83 ± 121.53	222.29 ± 115.70	215.37 ± 113.31	233.67 ± 127.92	279.21 ± 161.91	<0.001
Vitamin B12 (µg/d)	7.46 ± 0.15	6.67 ± 0.15	6.25 ± 0.15	5.82 ± 0.15	5.58 ± 0.15	<0.001	7.19 ± 0.16	6.19 ± 0.16	5.60 ± 0.16	5.42 ± 0.16	4.94 ± 0.16	<0.001
Folate (µg/d)	741.80 ± 5.30	730.09 ± 5.29	749.77 ± 5.30	741.24 ± 5.29	758.08 ± 5.33	<0.001	711.54 ± 5.41	707.57 ± 5.40	713.07 ± 5.41	718.50 ± 5.40	730.31 ± 5.44	<0.001
Phosphorus (mg/d)	1370.59 ± 381.51	1256.11 ± 388.85	1229.30 ± 373.65	1237.71 ± 366.12	1384.94 ± 346.16	<0.001	1335.94 ± 383.15	1190.35 ± 364.81	1163.85 ± 375.47	1171.86 ± 364.59	1325.26 ± 371.50	<0.001
Potassium (mg/d)	4986.37 ± 1198.06	3882.09 ± 980.42	3417.17 ± 941.57	3120.22 ± 945.85	2984.40 ± 868.56	<0.001	4992.59± 1276.18	3775.89 ± 931.66	3314.57 ± 941.48	2993.56 ± 910.07	2880.18 ± 897.30	<0.001
Calcium (mg/d)	1259.74 ± 424.31	1190.94 ± 433.45	1235.44 ± 449.91	1253.31 ± 442.35	1457.27 ± 468.65	<0.001	1229.67 ± 426.45	1146.76 ± 423.87	1184.55 ± 446.96	1231.08 ± 455.33	1436.68 ± 514.13	<0.001
Magnesium (mg/d)	436.26 ± 105.30	371.28 ± 101.50	349.65 ± 101.12	337.60 ± 97.09	357.36 ± 92.69	<0.001	429.29 ± 117.18	354.66 ± 98.24	332.16 ± 97.70	321.35 ± 94.83	343.60 ± 96.02	<0.001
Sodium (mg/d)	4302.67 ± 1466.07	4218.18 ± 1409.18	4264.09 ± 1432.23	4341.54 ± 1385.93	4867.51 ± 1438.86	<0.001	4365.69 ± 1720.51	4107.00 ± 1478.26	4150.44 ± 1522.62	4315.02 ± 1465.19	4800.86 ± 1475.00	<0.001
Sodium/potassium	0.88 ± 0.28	1.09 ± 0.31	1.27 ± 0.38	1.43 ± 0.38	1.70 ± 0.51	<0.001	0.89 ± 0.30	1.09 ± 0.31	1.27 ± 0.41	1.48 ± 0.43	1.74 ± 0.54	<0.001

Multivariable-adjusted OR for MetS and its components across NEAP and PRAL quintiles are presented in Table 4. Adherence to a diet with a high DAL (PRAL and NEAP) was not associated with increased odds of MetS in the crude or adjusted models. Among the components of MetS, in women, higher NEAP scores were associated with an increased odds of hypertriglyceridemia after adjusting for age, energy intake, physical activity, education, marital status, SES, home ownership, fatty liver disease, depression, thyroid disease, calcium supplementation, and calcium plus vitamin D supplementation in model I (OR: 1.54, 95% CI: 1.007-2.36, p trend = 0.02), and after further adjustment for BMI in the fully adjusted model (OR: 1.55, 95% CI: 1.01-2.40, p trend = 0.01). In the crude model, women in the bottom NEAP quintile were more likely to have elevated HDL-C than those in the top quintile (OR: 1.26, 95% CI: 1.01-1.56, p trend = 0.06). This association was still significant and even stronger after taking potential confounders into account (OR: 1.42, 95% CI: 1.001-2.03, p trend = 0.01 for model I; OR: 1.44, 95% CI: 1.009-2.06, p trend = 0.01 for model II). In addition, women in the highest PRAL quintile had greater odds of having elevated HDL-C than those in the fourth quintile in model I and the fully adjusted model (OR: 1.54, 95% CI: 1.08-2.19, p trend = 0.06 for model I; OR: 1.56, 95% CI: 1.10-2.23, p trend = 0.06 for model II).

**Table 4:** Odds ratios and 95% confidence intervals for prevalence of metabolic syndrome and its components across quintiles of PRAL and NEAP scores Note: Data are presented as odds ratios and 95% confidence intervals. MetS and its components are defined according to the Adult Treatment Panel III (ATP III) criteria. Abbreviations: PRAL, potential renal acid load; NEAP, net endogenous acid production; LDL-C, low-density lipoprotein cholesterol; HDL- C, high-density lipoprotein cholesterol; FBS, fasting blood sugar. ^a^ Obtained with binary logistic regression. ^b^ Adjusted for age, energy intake, physical activity, education, marital status, socioeconomic status, home ownership, fatty liver disease, depression, thyroid disease, calcium supplementation, and calcium plus vitamin D supplementation. ^c^ Additionally adjusted for BMI. ^d^ Central obesity was defined as waist circumference >102 cm in men and >88 cm in women. ^e^ Hypertension was defined as systolic blood pressure ≥ 130 mm Hg and/or diastolic blood pressure ≥ 85 mm Hg. ^f^ Low HDL-C was defined as HDL-C <50 mg/dL for women and <40 mg/dL for men. ^g^ Hyperglycemia was defined as FBS ≥ 110 mg/dL. ^h^ Hypertriglyceridemia was defined as triglycerides >150 mg/dL.

	Quintiles of PRAL					p trend^a^	Quintiles of NEAP					p trend^a^
Models	1	2	3	4	5		1	2	3	4	5	
Men												
Range		-23.81, -12.95	-12.95, -5.15	-5.13, 4.19	>4.19		<29.77	29.77, 37.26	37.30, 44.32	44.33, 53.52	>53.52	
n	637	638	638	638	638		569	643	644	658	675	
Metabolic syndrome												
Crude	1	0.82 (0.53-1.26)	0.71 (0.45-1.11)	1.04 (0.69-1.57)	0.77 (0.50-1.20)	0.61	1	0.86 (0.64-1.14)	1.03 (0.78-1.36)	0.91 (0.69-1.22)	1.01 (0.76-1.34)	0.92
Model ɪ^b^	1	0.54 (0.25-1.15)	0.68 (0.34-1.38)	1.24 (0.64-2.38)	1.11 (0.57-2.14)	0.21	1	0.68 (0.33-1.40)	0.77 (0.38-1.53)	1.004 (0.52-1.92)	1.07 (0.56-2.04)	0.55
Model ɪɪ^c^	1	0.52 (0.23-1.16)	0.67 (0.31-1.42)	0.88 (0.43-1.79)	0.93 (0.46-1.87)	0.71	1	0.30 (0.13-0.73)	0.57 (0.27-1.24)	1.04 (0.51-2.12)	0.81 (0.39-1.68)	0.4
Central obesity^d^												
Crude	1	0.88 (0.60-1.28)	0.91 (0.63-1.32)	0.89 (0.61-1.30)	0.86 (0.59-1.26)	0.51	1	1.15 (0.77-1.70)	1.05 (0.70-1.56)	1.04 (0.70-1.55)	1.03 (0.69-1.53)	0.91
Model ɪ	1	0.89 (0.48-1.64)	0.91 (0.50-1.66)	0.86 (0.47-1.59)	1.19 (0.67-2.12)	0.62	1	0.96 (0.52-1.76)	0.90 (0.49-1.65)	0.89 (0.47-1.67)	1.17 (0.64-2.12)	0.68
Model ɪɪ	1	0.48 (0.17-1.33_	0.81 (0.32-2.06)	0.63 (0.24-1.63)	0.67 (0.27-1.67)	0.59	1	0.36 (0.13-0.99)	0.59 (0.23-1.53)	0.44 (0.17-1.15)	0.79 (0.33-1.19)	0.84
Hypertension^e^												
Crude	1	1.03 (0.77-1.38)	0.80 (0.59-1.09)	0.90 (0.67-1.12)	0.91 (0.67-1.23)	0.33	1	0.77 (0.57-1.05)	0.85 (0.63-1.15)	0.80 (0.59-1.08)	0.88 (0.65-1.18)	0.56
Model ɪ	1	0.85 (0.53-1.36)	0.81 (0.51-1.29)	0.81 (0.50-1.30)	0.60 (0.60-1.53)	0.75	1	0.59 (0.36-0.97)	0.84 (0.53-1.34)	0.95 (0.59-1.52)	0.85 (0.52-1.37)	0.88
Model ɪɪ	1	0.79 (0.49-1.28)	0.78 (0.48-1.26)	0.76 (0.47-1.24)	0.88 (0.55-1.41)	0.55	1	0.55 (0.33-0.91)	0.82 (0.51-1.31)	0.90 (0.55-1.45)	0.76 (0.46-1.23)	0.82
Low HDL-C^f^												
Crude	1	0.97 (0.77-1.22)	0.95 (0.75-1.20)	0.98 (0.78-1.24)	0.95 (0.75-1.20)	0.74	1	0.90 (0.71-1.14)	0.92 (0.73-1.17)	0.83 (0.65-1.05)	0.95 (0.75-1.20)	0.51
Model ɪ	1	1.06 (0.74-1.53)	0.98 (0.69-1.40)	1.04 (0.73-1.48)	0.91 (0.63-1.30)	0.62	1	0.87 (0.60-1.25)	1.03 (0.72-1.47)	0.83 (0.57-1.20)	0.99 (0.69-1.43)	0.91
Model ɪɪ	1	1.03 (0.72-1.49)	0.98 (0.69-1.40)	1.02 (0.71-1.45)	0.88 (0.61-1.27)	0.53	1	0.86 (0.60-1.24)	1.03 (0.72-1.47)	0.81 (0.56-1.18)	0.96 (0.66-1.39)	0.76
Hyperglycemia^g^												
Crude	1	0.73 (0.36-1.46)	0.89 (0.46-1.072)	0.78 (0.39-1.55)	0.83 (0.43-1.64)	0.69	1	2.06 (0.97-4.37)	1.14 (0.49-2.63)	1.56 (0.71-3.42)	1.44 (0.65-3.11)	0.79
Model ɪ	1	1.24 (0.36-4.29)	0.81 (0.21-3.19)	1.97 (0.63-6.12)	1.54 (0.47-5.05)	0.29	1	4.29 (0.87-20.97)	1.98 (0.35-11.16)	5.68 (1.18-27.25)	3.81 (0.75-19.29)	0.11
Model ɪɪ	1	1.06 (0.30-3.80)	0.83 (0.21-3.30)	2.02 (0.63-6.46)	1.36 (0.41-4.57)	0.33	1	3.68 (0.72-18.64)	2.007 (0.34-11.58)	5.89 (1.19-28.99)	3.26 (0.62-17.05)	0.54
Hypertriglyceridemia^h^												
Crude	1	0.82 (0.64-1.05)	0.96 (0.76-1.22)	0.95 (0.75-1.21)	0.82 (0.64-1.04)	0.35	1	0.80 (0.63-1.03)	0.88 (0.68-1.12)	1.01 (0.79-1.29)	0.80 (0.63-1.03)	0.49
Model ɪ	1	0.80 (0.53-1.20)	0.91 (0.62-1.34)	1.03 (0.70-1.51)	1.06 (0.72-1.56)	0.45	1	0.84 (0.56-1.27)	0.96 (0.65-1.44)	1.05 (0.70-1.57)	1.25 (0.74-1.87)	0.13
Model ɪɪ	1	0.72 (0.47-1.10)	0.89 (0.59-1.34)	0.96 (0.64-1.43)	0.97 (0.65-1.45)	0.68	1	0.79 (0.51-1.21)	0.95 (0.63-1.44)	0.99 (0.65-1.50)	1.13 (0.75-1.70)	0.31
Women												
Range		-25.76, -14.92	-14.89, -6.63	-6.53, 1.90	>1.90		<29.77	29.80, 37.26	37.31, 44.32	44.33, 53.52	>53.54	
n	633	634	633	634	633		702	628	628	613	596	
Metabolic syndrome												
Crude	1	0.79 (0.59-1.06)	0.82 (0.62-1.10)	0.88 (0.66-1.16)	0.92 (0.69-1.22)	0.81	1	0.86 (0.64-1.14)	1.03 (0.78-1.36)	0.91 (0.69-1.22)	1.01 (0.76-1.34)	0.8
Model ɪ	1	0.88 (0.56-1.37)	1.17 (0.77-1.79)	1.29 (0.84-2.001)	1.03 (0.66-1.62)	0.37	1	0.77 (0.49-1.20)	0.92 (0.59-1.44)	1.09 (0.71-1.67)	0.89 (0.58-1.38)	0.82
Model ɪɪ	1	0.76 (0.48-1.22)	0.91 (0.57-1.44)	1.07 (0.68-1.68)	0.88 (0.56-1.38)	0.89	1	0.87 (0.55-1.39)	1.18 (0.76-1.83)	1.34 (0.85-2.11)	1.03 (0.65-1.64)	0.37
Central obesity												
Crude	1	1.06 (0.83-1.36)	0.91 (0.71-1.16)	0.74 (0.59-0.94)	0.98 (0.77-1.25)	0.15	1	0.94 (0.74-1.19)	0.92 (0.72-1.17)	0.68 (0.54-0.86)	0.89 (0.70-1.14)	0.04
Model ɪ	1	1.14 (0.76-1.71)	0.78 (0.42-1.17)	0.87 (0.59-1.28)	1.29 (0.86-1.96)	0.69	1	0.74 (0.50-1.09)	0.81 (0.55-1.21)	0.78 (0.52-1.16)	0.97 (0.64-1.47)	0.87
Model ɪɪ	1	0.61 (0.34-1.09)	0.70 (0.38-1.28)	0.83 (0.45-1.52)	1.11 (0.60-2.06)	0.58	1	1.03 (0.55-1.91)	0.59 (0.31-1.11)	0.88 (0.47-1.64)	1.48 (0.79-2.78)	0.4
Hypertension												
Crude	1	0.74 (0.54-1.03)	0.80 (0.58-1.10)	0.95 (0.70-1.29)	0.87 (0.64-1.19)	0.88	1	0.66 (0.48-0.91)	0.76 (0.56-1.04)	0.95 (0.70-1.28)	0.81 (0.59-1.11)	0.75
Model ɪ	1	0.68 (0.40-1.17)	0.80 (0.47-1.35)	0.88 (0.53-1.47)	1.05 (0.64-1.72)	0.61	1	0.65 (0.38-1.11)	0.84 (0.50-1.40)	1.003 (0.60-1.65)	0.96 (0.58-1.59)	0.7
Model ɪɪ	1	0.69 (0.40-1.18)	0.79 (0.46-1.35)	0.85 (0.51-1.43)	1.06 (0.64-1.73)	0.65	1	0.64 (0.38-1.10)	0.83 (0.50-1.39)	0.97 (0.58-1.63)	0.96 (0.58-1.60)	0.72
Low HDL-C												
Crude	1	1.12 (0.90-1.39)	1.12 (0.90-1.40)	1.13 (0.91-1.41)	1.19 (0.96-1.49)	0.14	1	1.07 (0.86-1.33)	1.24 (1.006-1.54)	1.06 (0.85-1.32)	1.26 (1.01-1.56)	0.06
Model ɪ	1	1.27 (0.89-1.80)	1.55 (1.08-2.23)	1.54 (1.08-2.19)	1.30 (0.91-1.84)	0.06	1	1.16 (0.82-1.62)	1.63 (1.15-2.31)	1.46 (1.02-2.08)	1.42 (1.001-2.03)	0.01
Model ɪɪ	1	1.27 (0.89-1.81)	1.58 (1.09-2.28)	1.56 (1.10-2.23)	1.30 (0.91-1.86)	0.06	1	1.18 (0.83-1.66)	1.66 (1.17-2.35)	1.50 (1.05-2.15)	1.44 (1.009-2.06)	0.01
Hyperglycemia												
Crude	1	0.75 (0.39-1.46)	1.05 (0.57-1.93)	1.002 (0.54-1.85)	0.86 (0.45-1.62)	0.95	1	0.92 (0.49-1.70)	0.87 (0.46-1.63)	1.15 (0.64-2.08)	0.76 (0.39-1.48)	0.73
Model ɪ	1	1.05 (0.68-1.62)	0.99 (0.63-1.55)	1.23 (0.81-1.90)	1.37 (0.90-2.08)	0.7	1	0.93 (0.35-2.46)	0.79 (0.28-2.23)	1.64 (0.67-4.01)	0.44 (0.13-1.48)	0.6
Model ɪɪ	1	0.44 (0.14-1.37)	0.84 (0.32-2.24)	1.03 (0.41-2.55)	0.48 (0.16-1.04)	0.6	1	0.95 (0.35-2.56)	0.80 (0.28-2.30)	1.60 (0.64-4.01)	0.42 (0.12-1.45)	0.14
Hypertriglyceridemia												
Crude	1	1.03 (0.79-1.35)	0.87 (0.67-1.15)	1.03 (0.79-1.35)	1.14 (0.88-1.48)	0.37	1	0.99 (0.76-1.29)	1.08 (0.83-1.40)	1.03 (0.79-1.35)	1.20 (0.93-1.56)	0.17
Model ɪ	1	0.44 (0.14-1.36)	0.86 (0.33-2.27)	1.10 (0.45-2.70)	0.50 (0.17-1.45)	0.1	1	1.07 (0.69-1.65)	1.34 (0.88-2.05)	1.41 (0.91-2.18)	1.54 (1.007-2.36)	0.02
Model ɪɪ	1	1.04 (0.67-1.62)	0.98 (0.62-1.55)	1.24 (0.80-1.91)	1.37 (0.89-2.09)	0.1	1	1.07 (0.69-1.66)	1.35 (0.88-2.07)	1.45 (0.93-2.25)	1.55 (1.01-2.40)	0.01

Lastly, in model I, men in the fourth NEAP quintile had 5.68-fold greater odds of hyperglycemia (OR: 5.68, 95% CI: 1.18-27.25, p trend = 0.11). Similar results were found after further adjustment for BMI in the fully adjusted model (OR: 5.89, 95% CI: 1.19-28.99, p trend = 0.54).

## Discussion

In this cross-sectional study, even after adjusting for potential confounders, adherence to a diet with a high DAL was not associated with increased odds of MetS in Iranian adults. Among the components of MetS, a significant association was observed between higher DAL (PRAL and NEAP) scores and increased odds of low HDL-C in women. In addition, higher DAL (NEAP) scores were significantly associated with greater odds of having hypertriglyceridemia. In men, moderate DAL (NEAP) was significantly associated with an increased odds of hyperglycemia.

The PRAL and NEAP scores are used to measure DAL from dietary intakes. Meat, poultry, fish, dairy, eggs, grains, and alcohol have more acid precursors and are related to a higher DAL. In contrast, most fruits, nuts, legumes, potatoes, and vegetables have more alkaline precursors and are related to a lower DAL [20]. Protein, sulfur, and phosphate are the acidic precursors, whereas alkaline precursors include calcium, potassium, and magnesium [4,20]. Thus, DAL may vary among different populations with different dietary habits and cultures. For example, the median NEAP score of the western dietary pattern rich in acid-forming foods is 34 to 76 mEq/d, whereas, for a vegan diet, the NEAP score is 7.26 mEq/d [20].

In the present study, neither PRAL nor NEAP was associated with MetS. In line with these findings, in a cross-sectional survey of 1430 Iranian adults [14], no significant association was found between DAL and MetS. Moreover, another cross-sectional study of 371 Iranian women (20-50 years old) revealed no significant association between DAL and MetS [12]. Research by Tangestani et al. did not reveal a statistically significant association between PRAL score and MetS in 246 Iranian women with overweight or obesity [16]. However, two Japanese cross-sectional studies contradict these findings [13,15]. Iwase et al. reported that increased DAL (both PRAL and NEAP) was associated with the prevalence of MetS in 260 Japanese patients with type 2 diabetes [15]. In addition, another study of Japanese participants (35-69 years old) found that higher NEAP was positively associated with the prevalence of MetS [13].

One reason for this discrepancy might be the between-population differences in mean DAL. As in most studies [12,26-28] of Iranians with transitional dietary patterns, PRAL (mean: −11.63 mEq/d; median: −9.78) and NEAP (mean: 42.30 mEq/d; median: 40.74) values in the present study are much lower than the mean values reported for the western dietary pattern. Furthermore, differences in the study population, sociodemographic characteristics, the method of dietary intake assessment, criteria to identify MetS, behavioral and lifestyle factors (such as dietary patterns and habits), as well as the number and type of confounding factors controlled for in the analysis, may also explain the inconsistent results [11,27].

Although we found no association between DAL and MetS, the potential mechanism that links higher DAL with an increased odds of MetS may be a decrease in insulin sensitivity due to chronic metabolic acidosis induced by long-term consumption of an acidogenic diet. Increased DAL causes increased cortisol production, decreased urinary citrate secretion, and increased magnesium excretion, resulting in reduced insulin sensitivity [3].

In addition, we found that a higher DAL was associated with greater odds of having hypertriglyceridemia and low HDL-C in women. In line with the present findings, Bahadoran et al. reported that a higher DAL (PRAL and protein/potassium ratio (Pro/K)) was associated with higher TG and lower HDL-C [26]. Moreover, in a cross-sectional study by Kucharska et al. of 6170 Polish participants aged >20 years, NEAP was positively associated with TG and negatively associated with HDL-C [29]. In addition, other studies [11,12] showed that DAL was positively associated with TG. In contrast, some cross-sectional studies found no significant association between DAL and TG or HDL-C [12,15,28-30].

Little knowledge is available on the mechanisms behind alterations in TG and HDL-C levels associated with higher DAL scores. However, systemic metabolic acidosis results in the relocation of free fatty acid from adipocytes to the blood, which may elevate blood cortisol levels and stimulate lipolytic activity [31]. Increased TG content with a high acid load may increase very low-density lipoprotein and TG concentrations in the liver [32].

The present study found an association between moderate DAL (NEAP) and increased odds of hyperglycemia in men. In line with this finding, Haghighatdoost et al. demonstrated that higher DAL (PRAL and Pro/K) was significantly associated with higher HbA1C, but that PRAL score was inversely associated with FBS [30]. Similarly, Kucharska et al. reported that the NEAP score was positively associated with the prevalence of T2DM and FBS [29]. In contrast, Amodu et al. found negative associations between higher NEAP scores and the prevalence of T2DM [33].

The lack of a significant direct association between the last NEAP quintile and hyperglycemia may be because men in this quintile consumed high amounts of protein-rich foods, which, in addition to containing acidic precursors, can reduce the glycemic load and glycemic index of the diet [34]. In addition, the number of patients with MetS was low in the last quintile.

A major strength of the present study is the large sample size, which made it possible to adjust for multiple covariates. A second strong point is that we considered calcium supplements (a crucial, commonly used alkaline-forming supplement in Iran [35]), in the adjusted analysis. Third, the data on dietary intakes were obtained with a validated, reliable FFQ. Lastly, our analysis excluded participants with a history of disorders that can affect renal function, which plays a crucial role in acid-base balance [36].

The present findings should nonetheless be interpreted in the context of certain limitations. First, because of the cross-sectional study design, the associations reported here do not necessarily indicate causation. Second, the PRAL and NEAP score estimations were based on self-reported dietary intake rather than objective assessment. There is a strong correlation between DAL scores and measured acid load based on 24-hour urine collection [4,20]. However, it is unclear whether the source of the observed associations is a high intake of anti-MetS foods (fruits and vegetables) and nutrients (potassium, calcium, magnesium) reflected in the DAL scores or a high DAL per se. Third, some observations may be biased by residual confounding or unmeasured factors. Fourth, this study was conducted in a developing country where dietary habits are changing; thus, our results may not be generalizable to other regions.

Although we considered calcium supplement intakes in adjusted models, we propose to include the amount of calcium in the supplements in the PRAL equation to achieve a more accurate estimation of DAL. In addition, dietary fat and the proportion of fatty acids might be related to the development of metabolic abnormalities [37]; however, they are not addressed by DAL scores. Moreover, although an increase in dietary protein might enhance weight reduction [38], an increase in total protein intake is accompanied by an increase in DAL [20], and they might cover the effects of each other in assessing the association between DAL and some metabolic risk factors (particularly obesity-related factors). Thus, in future studies, it might be helpful to consider the defects of these scores and find a way to cope with them.

## Conclusions

Our findings suggest that a higher DAL was inversely associated with HDL-C and directly associated with serum TG levels in women. Moreover, there was a positive association between the fourth quintile of NEAP and FBS in men. However, even after adjusting for potential confounders, there was no significant association between DAL and the odds of MetS in the sample of Iranian adults studied here. Longitudinal studies are warranted to shed further light on the replicability of these findings.
